# Lyre sign – Where schwannoma mimics a carotid body tumour

**DOI:** 10.4102/sajr.v29i1.3072

**Published:** 2025-05-12

**Authors:** Fatin N. Sahar, Hilwati Hashim, Norliana D. Mohamad Ali, Masaany Mansor, Yin P. Wong

**Affiliations:** 1 Department of Radiology, Faculty of Medicine, Universiti Teknologi MARA, Sungai Buloh, Malaysia; 2 Department of Otorhinolaryngology, Faculty of Medicine, Universiti Teknologi MARA, Sungai Buloh, Malaysia; 3 Department of Pathology, Faculty of Medicine, Universiti Kebangsaan Malaysia, Kuala Lumpur, Malaysia

**Keywords:** schwannoma, neck neoplasms, magnetic resonance imaging, carotid body tumor, parapharyngeal neoplasms

## Abstract

Nerve sheath tumours of the sympathetic chain are extremely rare and present a diagnostic challenge. A 42-year-old female presented with asymptomatic left cervical swelling. Imaging with sonography, CT, MR and angiography demonstrated a lesion splaying the carotid bifurcation, the lyre sign. Following surgical excision, histology revealed a schwannoma. Imaging features of carotid body tumours and parapharyngeal schwannomas can be similar, as both can cause splaying of the carotid bifurcation. The differential diagnosis of such tumours and their management are discussed.

**Contribution:**

This article highlights another cause of the lyre sign on radiological imaging besides carotid body tumours.

## Introduction

Lyre sign refers to the splaying of the internal carotid artery (ICA) and external carotid artery (ECA) at the carotid bifurcation secondary to a mass, visualised on CT, MRI and/or digital subtraction angiography (DSA). It is thought to be a sign suggestive of a carotid body tumour. However, schwannomas from the cervical sympathetic chain and lower cranial nerves can develop near the carotid bifurcation and display similar vascular displacement. This report discusses a patient with a left parapharyngeal neck schwannoma causing splaying of the carotid bifurcation, mimicking a carotid body tumour.

## Patient presentation

A 42-year-old female, with no prior medical illness, presented with a 1-year history of an enlarging left neck swelling, associated with occasional pulsating pain and difficulty turning her head. Her father and mother had been diagnosed with nasopharyngeal and colon cancers, respectively, and succumbed to their malignancies. There was no other relevant family history.

Upon assessment, her vital signs were normal. There was a firm, non-tender and immobile swelling in the left anterolateral cervical region. Flexible nasopharyngoscopy showed minimal left lateral pharyngeal wall medialization at the level of the oropharynx.

## Imaging findings

**FIGURE 1:**
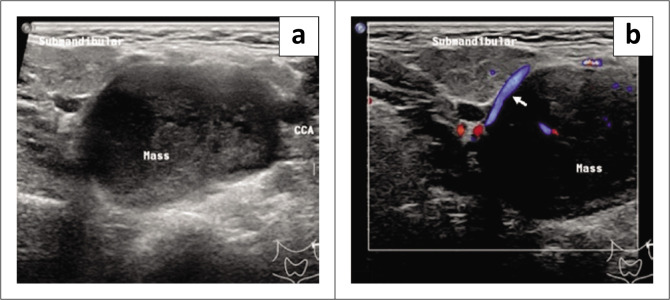
(a) Transverse greyscale ultrasound of the neck revealed a well-defined hypoechoic mass lateral and inferior to the left submandibular gland and medial to the left common carotid artery. (b) Transverse Doppler ultrasound of neck. Note minimal internal vascularity (white arrow) demonstrated on colour Doppler ultrasound.

Ultrasound of the neck revealed a well-defined hypoechoic mass with cystic components and internal microcalcifications, inferolateral to the left submandibular gland. Minimal internal vascularity was demonstrated (Ultrasound with Colour Doppler/Epiq 5G, Philips) (Figure 1).

**FIGURE 2:**
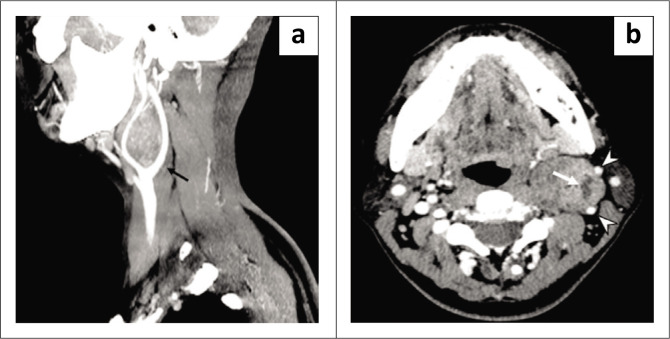
(a) Contrast-enhanced CT neck sagittal view in maximal intensity projection (MIP) showing a well-defined, heterogeneously enhancing mass at the left carotid bifurcation resulting in splaying of the left internal and external carotid arteries in keeping with lyre sign (black arrow). (b) Contrast-enhanced CT neck axial view. Note the internal cystic component (white arrow), lateral displacement of the left internal and external carotid arteries (white arrowheads) with non-visualisation of the left internal jugular vein likely due to compression by the mass.

Contrast-enhanced neck CT demonstrated a well-defined, heterogeneously enhancing mass with a necrotic centre measuring 3.4 cm (AP) × 3.4 cm (W) × 4.7 cm (CC) at the left carotid bifurcation. The mass resulted in splaying and displacement of the left ECA anterolaterally and the left ICA posterolaterally, with no clear fat plane between them; the vessels were both patent. Laterally, the mass compressed the left internal jugular vein (IJV) (Somatom Definition AS+, Siemens Medical Solutions) (Figure 2).

**FIGURE 3:**
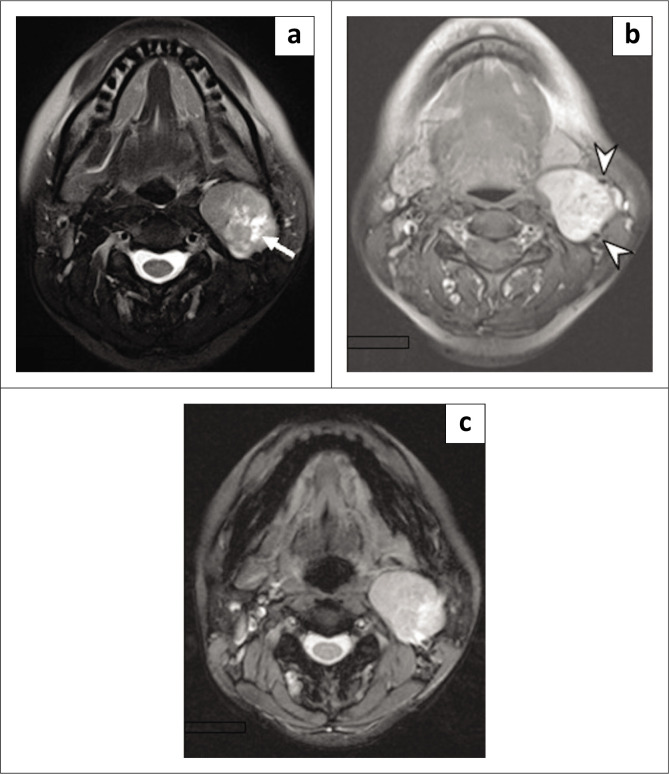
(a) Axial MRI neck T2W fat-saturated image showing a heterogeneously hyperintense soft tissue mass at the left carotid bifurcation with an internal cystic component (white arrow). (b) Axial T1W fat-saturated post-gadolinium image showing splaying of the left internal and external carotid arteries (white arrowheads). (c) Axial FL 2D. Note lack of vascularity within the tumour.

MRI of the neck displayed the mass as isointense to muscle on T1-weighted imaging, heterogeneously hyperintense on T2-weighted imaging and heterogeneously enhancing on contrast-enhanced T1-weighted fat-suppression imaging (Figure 3). Blooming artefacts on T2-dimensional fast-low-angle-shot (FL 2D) image suggested the presence of calcifications or haemorrhagic components. No flow voids were seen within the mass (Area 1.5T, Siemens Medical Solutions) (Figure 3).

## Pathological examination, surgical management and outcome

Ultrasound-guided fine needle aspiration cytology (FNAC) of the mass revealed moderately cellular smears, demonstrating clusters of benign spindle cells on a background of myxoid matrix. The spindle cells had elongated, spindle-shaped nuclei and ill-defined cytoplasm. No cytological atypia or mitosis was found. A cytological interpretation of a benign spindle cell neoplasm was rendered. The sample was insufficient for cell block preparation and immunocytochemistry assessment. Tumour resection was hence suggested.

A left transcervical surgical resection of the neck mass was performed by the neurosurgical team. Intraoperatively, a well-encapsulated solid mass with a smooth surface was identified anteromedial to the left sternocleidomastoid muscle, between the left ICA and ECA, splaying these arteries. The left vagus nerve was identified deep to the tumour. The left hypoglossal nerve and descending segment of ansa cervicalis were located, and all the identified nerves were preserved prior to the removal of the tumour. A post-operative diagnosis of left carotid body tumour was suggested.

**FIGURE 4:**
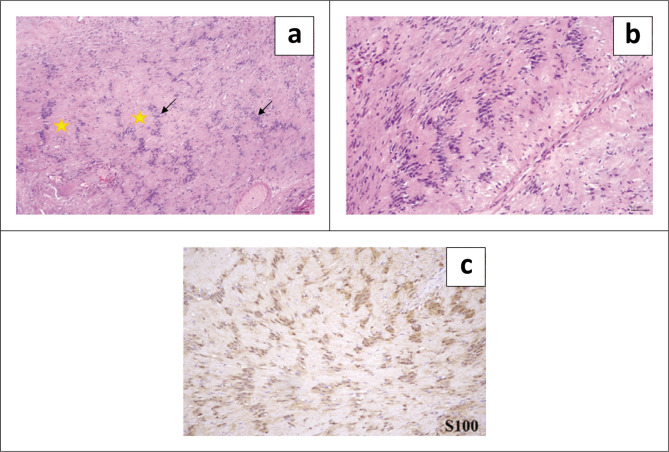
Histological examination of the parapharyngeal neck schwannoma. (a) The lesion is composed of hypercellular Antoni A areas (black arrows) and hypocellular Antoni B areas (yellow stars) (H&E, ×40). (b) Higher magnification shows neoplastic cells displaying elongated spindle to oval nuclei, eosinophilic cytoplasm with ill-defined cellular borders (H&E, ×200). (c) Immunohistochemically, the neoplastic cells are positive to S100 (S100, ×200).

Histological examination of the left neck mass revealed a well-circumscribed, encapsulated biphasic lesion composed of compact hypercellular Antoni A and myxoid hypocellular Antoni B areas. The neoplastic cells exhibited elongated spindle cells with tapered ends, dense nuclear chromatin pattern and eosinophilic cytoplasm with ill-defined cellular borders. Prominent Verocay bodies showing nuclear palisading around fibrillary processes, a few thick-walled vessels and foci of haemorrhages were also present. Mitosis was inconspicuous, and no tumour necrosis was observed. Immunohistochemically, the neoplastic cells were positive for S100. A histological diagnosis of schwannoma was made (Figure 4).

Post-operatively, the patient’s symptoms resolved, and she remains under the care of the Department of Neurosurgery for surveillance. No tumour recurrence has been documented.

## Discussion

The lyre sign refers to splaying of the ICA and ECA at the carotid bifurcation, caused by a mass located between them. The splayed vessels resemble a lyre, a U-shaped musical instrument used in ancient Greece. This appearance is classically a distinctive feature of carotid body tumours.^1,2,3,4,5^ Although splaying of the carotid bifurcation on radiological imaging helps to differentiate carotid body tumours from other post-styloid parapharyngeal masses, cervical sympathetic chain and vagal schwannomas may present with similar vascular displacement.^1,2,3,4,5^

Schwannomas are common between the ages of 30 and 50 years, with no gender predisposition.^2^ Schwannomas in the parapharyngeal space arise from the cervical sympathetic chain and vagus nerve. The cervical sympathetic chain runs along the posterior and medial borders of the carotid sheath. Consequently, cervical sympathetic chain schwannomas may cause displacement of the ICA, ECA and IJV in a similar direction anterolaterally, without separating or encasing these vessels.^1,5^ Owing to its origin, vagal schwannomas typically separate the ICA and IJV and cause anteromedial displacement of the ICA.^5^ Schwannomas are commonly hypovascular, while hypervascular schwannomas are rare and difficult to distinguish from carotid body tumours.^1,2^ Ultrasound colour Doppler may demonstrate some degree of vascularity within the tumours. The soft tissue tumour heterogeneity is relatively common, because of cystic degeneration or a relative hypocellular area adjacent to densely cellular or collagenous regions.^1^ On CT imaging, schwannomas are hypodense with mild contrast enhancement and may have cystic areas within. The tumours are iso- to hypointense on T1-weighted images and hyperintense on T2-weighted images on MRI.^6^

Carotid body tumours are uncommon, although they represent most of the head and neck paragangliomas. The tumours are common in young people, with a female preponderance.^1,2,3,4,5^ Carotid body tumours develop from neural crest cells located at the posteromedial aspect of the carotid bifurcation.^4,7^ As the carotid body tumour grows, it causes splaying of the ICA and ECA, producing the lyre sign.^1,2,3,4,5^ Growing tumours at the carotid bifurcation frequently involve the vagus nerve and sympathetic chains. The vagus nerve travels through the neck and passes within the carotid sheath between the IJV and CCA, while the sympathetic chains run deep to the carotid sheath between the IJV and ICA, alongside the vertebral arteries in the neck.^8^

Tumours that arise at or near the carotid bifurcation are also referred to as post-styloid tumours as they are located posterior to the styloid process of the temporal bone. Other post-styloid lesions are very rare and may originate from soft tissue, nerves or vessels. These include neurofibromas, lymphoma, metastatic tumours and carotid artery aneurysms.^9^

Radiological imaging, particularly CT and MRI, are helpful in differentiating these lesions as they exhibit distinct imaging characteristics. Carotid body tumours are well-defined, avidly enhancing hypervascular lesions, typically located at the carotid bifurcation, causing splaying of the arteries. These tumours exhibit a sand-and-pepper appearance on MRI because of the presence of vascular flow voids. While schwannomas are well-circumscribed homogeneously enhancing lesions, neurofibromas demonstrate heterogeneous enhancement and are usually multiple. Lymphomas tend to be large, infiltrative, homogeneous, non-vascular lesions. Metastatic tumours are heterogeneously enhancing with irregular borders and may be associated with adjacent osseous destruction. Carotid artery aneurysms appear as soft tissue masses with flow voids on MRI and often demonstrate central low attenuation on CT scans, representing thrombus.^9^

Based on the clinical and radiological findings in the presented case, a provisional diagnosis of a carotid body tumour and differential diagnosis of a schwannoma was made. The left neck mass was located at the left carotid bifurcation causing splaying of the left ICA and ECA as well as displacing these arteries, along with the left IJV in the same direction, creating a diagnostic dilemma between carotid body tumour and schwannoma. Internal vascularity and enhancement patterns are key features that help in distinguishing carotid body tumours from schwannomas. Hypervascular schwannomas are rare and difficult to distinguish from carotid body tumours. In contrast with carotid body tumour, no flow voids are seen in schwannomas.^1,2,3^ In this case, the left neck mass showed internal vascularity on ultrasound and heterogeneous enhancement on CT and MRI. Blooming artefacts were seen on the MRI, however, no flow voids were present in the mass.

Fine needle aspiration cytology offers limited presurgical advantage, as demonstrated in this case.^3^ Most FNACs of schwannomas furnish inconclusive results because of challenges in yielding adequate cellularity, owing to the dense stromal components and cystic degeneration.^3^

Definitive diagnosis is established through histopathological evaluation. Schwannomas display a characteristic histological appearance, consisting of two distinct components: a hypercellular area (Antoni A) and a loose myxoid area (Antoni B).^5^ The histology of schwannomas may sometimes be difficult to identify with haematoxylin and eosin stains alone. This entity can be confused with other benign spindle cell lesions such as neurofibroma and solitary circumscribed neuroma. Immunohistochemistry stains with S100, desmin and epithelial membrane antigen (EMA) are often helpful for the distinction,^7^ especially in cases with equivocal histomorphological features.

Complete surgical excision provides both a definitive diagnosis and curative treatment for carotid body tumours and parapharyngeal schwannomas.^2,5^ CT and MRI are helpful to delineate extension and to visualise its relationship with adjacent structures. As resection of carotid body tumour carries higher risk of intraoperative haemorrhage, preoperative angiography and embolisation are essential prior to resection. Radiotherapy is reserved for the elderly or unresectable cases.^2^ Prognosis is excellent for schwannomas, as the tumours are usually encapsulated and recurrence is very rare.^10^ Excision of vagal schwannomas is usually without complication, apart from the risk of ipsilateral vocal fold palsy in cases where the tumour is proximal to the recurrent laryngeal nerve. However, removal of sympathetic ganglia schwannomas can result in Horner’s syndrome. Focal recurrences of carotid body tumours have been reported in the literature, so close follow-up is crucial.^2^

## Conclusion

The radiological finding of the lyre sign is classically a distinguishing feature of carotid body tumours. However, it can also be observed in parapharyngeal schwannomas. Hypovascular tumours suggest schwannomas, while hypervascular tumours are suggestive of carotid body tumours.

Radiological imaging, along with histopathology and immunohistochemical staining, is helpful in clinching the diagnosis in these cases. Preoperative diagnosis is important in these post-styloid parapharyngeal masses.
